# Assessment of Humoral Immunogenicity of ChAdOx1 H5 HA Influenza Vaccine for Dairy Cattle

**DOI:** 10.3390/vaccines14080691

**Published:** 2026-08-12

**Authors:** Barbara Dema, Marta Ulaszewska, Alice Lilley, Abi Lofts, Roo Bhasin, Ruth Harvey, Piyada Supasa, Matěj Hlaváč, Susan J. Morris, Richard E. Booth, Alexander M. P. Byrne, Nicola Lewis, Alex McSloy, Sarah C. Gilbert

**Affiliations:** 1 Pandemic Sciences Institute, Nuffield Department of Medicine, University of Oxford, Oxford OX3 7TY, UK; 2 Worldwide Influenza Centre, Francis Crick Institute, London NW1 1AT, UK; 3 Centre for Human Genetics, Nuffield Department of Medicine, University of Oxford, Oxford OX3 7BN, UK; 4 Pathobiology and Population Sciences, Royal Veterinary College, London NW1 0TU, UK; 5 Biological Services Unit, Royal Veterinary College, London NW1 0TU, UK; 6 CAMS Oxford Institute, Chinese Academy of Medical Sciences & Peking Union Medical College, University of Oxford, Oxford OX3 7BN, UK

**Keywords:** avian influenza, vaccine, ChAdOx1, adenoviral vector, humoral immunity, IgA

## Abstract

Background/objectives: The emergence of highly pathogenic avian influenza A (H5N1) virus infections in dairy cattle in the United States revealed a novel mammalian host and a potential transmission pathway involving raw milk and dairy production systems. Sustained circulation of H5N1 in dairy herds is of concern because ongoing viral adaptation in mammals may increase the risk of efficient mammalian transmission and subsequent zoonotic spread. In response to this emerging threat, we developed a chimpanzee adenovirus (ChAd)-vectored vaccine expressing the haemagglutinin 5 antigen (H5HA) from the dairy cattle isolate A/dairy cattle/Texas. Methods: Lactating dairy cows were vaccinated by either intramuscular (N = 3) or intranasal administration (N = 3). H5HA clade 2.3.4.4b antibodies in cow’s milk and sera were evaluated by ELISA. Hemagglutination and neutralisation capacity were also evaluated. Results: IgG and IgA H5HA-specific antibodies were detected in serum and milk from parenterally vaccinated animals, demonstrating the induction of a systemic immune response. The neutralising antibody responses elicited were only detected in serum of cows vaccinated via the intramuscular route. Conclusions: These preliminary findings support the feasibility of ChAd-vectored vaccination as a strategy to induce humoral immunity in cattle against emerging H5N1 influenza A viruses. Cross-reactive antibody responses against both *A/dairy cattle/Texas/24-008749_001/2024* and *A/Ibis/Egypt/RLQP-229S/2022* support the capacity of the vaccine to recognise antigenically related H5N1 clade 2.3.4.4b viruses circulating in mammalian and avian reservoirs. Further studies of vaccine efficacy and the immunological mechanism of protection should now be undertaken with the aim of reducing viral transmission and milk-associated shedding in dairy herds.

## 1. Introduction

Highly pathogenic avian influenza (HPAI) H5N1 viruses, belonging to clade 2.3.4.4b, have been causing outbreaks since 1996 in birds and different mammals. Unprecedented geographical expansion since 2021 has resulted in extensive outbreaks in wild birds, poultry and mammalian species. Viruses from this lineage have been detected across Africa, Europe, Asia and the Americas. More recently, detections of H5N1 clade 2.3.4.4b influenza A virus infections in dairy cattle within the United States [1] have demonstrated that viable influenza A virus can be present in raw milk, revealing a potentially novel cow-to-cow transmission pathway that may involve the milking process and/or equipment, or other unknown transmission routes [2]. Lactating cows appear to be disproportionately affected, exhibiting decreased milk production and the secretion of abnormal milk characterised by increased viscosity and discolouration. This often necessitates milk disposal, resulting in substantial economic losses. Although the epidemiological and pathogenic consequences of this finding remain uncertain, it demonstrates the capacity of the virus to infect an additional domesticated, food-producing mammalian species, alongside confirmed infections in pigs and sheep. Farm-to-farm spread has been demonstrated in the United States via infected cattle. The sustained circulation of an avian-origin influenza A virus within mammalian hosts that maintain close contact with, and produce food products for humans, is of significant concern, as ongoing viral adaptation could facilitate efficient mammalian-to-mammalian transmission, thereby heightening the risk of a H5N1 influenza pandemic. Indeed, a total of 70 confirmed cases in humans have been reported in the United States since 2024, 60% being caused by exposure to dairy herds, and 35% related to poultry farms. Moreover, since 1997, 25 countries, including the United States, have reported a cumulative total of more than 1000 sporadic human infections with HPAI A/H5N1 viruses, and approximately 48% have died, showing the continued potential and dangerous global threat of an H5N1 pandemic [3].

Vaccination represents an important tool to limit viral transmission within dairy cattle, reduce viral shedding in milk, and reduce the economic and public health consequences associated with H5N1 HPAI outbreaks. Several H5 influenza vaccines have been developed in response to cattle influenza A virus infections [4,5,6,7]. However, very little is known about whether H5 influenza vaccines induce an immune response in cow’s milk, or even whether influenza A vaccines that may induce mucosal immunity can provide additional immunological benefits at the mammary glands and through the milk. A recent investigation that used an H5 inactivated vaccine or DNA vaccine showed that intramuscular immunisation induced protection in cattle after a high dose of challenge virus was inoculated directly into the mammary glands and prevented viral dissemination among cross-suckling calves [8]. These findings indicate that systemic immunisation can generate an effective immune response capable of protecting the mammary glands; however, the specific protective mechanism has not been explained and needs further investigation.

Adenoviral vectored vaccines have been proven to elicit a robust immune response against the encoded transgene. Adenoviral vectors have been tested in several clinical trials, in some cases leading to licensure. One widely recognised and notable example was the use of the replication-defective chimpanzee adenovirus ChAdOx1 nCov-19/AZD1222/Vaxzevria, during the COVID-19 pandemic, which demonstrated a good safety profile in addition to low manufacturing and distribution costs. [9]. Although adenoviral vectors have been widely investigated in veterinary medicine and have demonstrated strong immunogenicity and protective efficacy, only a few vaccines have been successfully licensed for commercial use. These include an oral adenoviral-vectored vaccine for control of sylvatic rabies in skunks and raccoons in Canada, and an adenoviral-vectored vaccine licensed by USDA against foot and mouth disease virus for pigs [10]. Several ChAdOx platform-based candidate vaccines are in advanced development for both human and veterinary applications, such as the Rift Valley fever vaccine (ChAdOx1 RVF), which has demonstrated protective efficacy in sheep, goats, and cattle [11,12].

In this study, we aimed to evaluate whether ChAdOx1 carrying the Haemagglutinin H5 from influenza *A/dairy cattle/Texas/24-008749_001/2024*, belonging to the HPAI clade 2.3.4.4b, ChAdOx1 H5-Texas, induced a humoral response in cattle by either intramuscular or intranasal vaccination, and assessed any vaccine-related adverse events. We demonstrate that both immunisation routes are well tolerated by cows. We show that intramuscular immunisation induces high titres of H5-specific IgA and IgG in sera and milk, detectable as early as 7 to 9 days after prime immunisation against two geographically distant H5N1 clade 2.3.4.4b viruses. Notably, anti-H5 antibody titres in serum were able to neutralise H5N1 virus in vitro, indicating the induction of functional antibodies. These preliminary findings support the further evaluation of ChAdOx1-H5-Texas as a promising candidate for the control of H5N1 in dairy cattle.

## 2. Materials and Methods

### 2.1. Chemicals and Plasticware

Unless specified otherwise, all culture media, reagents, and plasticware used in this work were supplied by Thermo Fisher Scientific (Altrincham, UK).

### 2.2. Influenza Virus Strains and Recombinant Proteins

The HA proteins of Influenza *A/dairy cattle/Texas/24-008749_001/2024*, Influenza *A/Michigan/90/2024*, and Influenza *A/American wigeon/South Carolina/22-000345-001/2021* are identical at the amino acid level (100% sequence identity). Therefore, these strains were used interchangeably according to reagent availability and standardised assay protocols.

### 2.3. Adenoviral Vaccine Vectors

A nucleotide sequence for haemagglutinin (HA) derived from avian influenza A Virus *A/Ibis/Egypt/RLQP-229S/2022* (H5N1) (GenBank accession number UXE55277), Clade 2.3.4.4b, was human codon optimised and runs of more than 4 consecutive bases removed before being synthesised and cloned into a Gateway^®^-compatible pENTR™ shuttle plasmid downstream of a modified human cytomegalovirus immediate early promoter containing intron A and a tetracycline-regulated operator sequence [13], and upstream of the bovine growth hormone polyadenylation signal by Twist Bioscience UK LTD (London, UK). The *A/Ibis/Egypt/RLQP-229S/2022* derived HA ORF was modified by site directed mutagenesis to introduce 6 amino acid substitutions (L120M, L131Q, T211I, V226A, I526V, and I548M) to generate the full-length HA ORF representative of the bovine H5HA of *A/dairy cattle/Texas/24-008749_001/2024* (GISAID accession no. EPI_ISL_19014384). Using the synthesised *A/Ibis/Egypt/RLQP-229S/2022* HA nucleotide sequence as a template, mutation-specific primers matched to the bovine H5 HA sequence were used to generate 4 PCR products using Phusion™ High-Fidelity DNA Polymerase (NEB). BamHI and EcoRI restriction enzyme sequences were included in the 5′ and 3′ ORF primers, respectively, for cloning purposes. PCR products were assembled into the plasmid pcDNA3.1 (Thermo Fisher Scientific, Altrincham, UK) between the BamHI and EcoRI restriction sites using Gibson assembly (New England Biolabs, Ipswich, MA, USA) to produce a seamless full-length bovine H5HA gene sequence. The sequence was verified by Sanger sequencing. The bovine H5HA ORF was subsequently cloned into a Gateway^®^-compatible pENTR™ shuttle plasmid using NEBuilder^®^ HiFi DNA Assembly Master Mix (New England Biolabs, Ipswich, MA, USA), placing the ORF downstream of a modified human cytomegalovirus immediate early promoter containing intron A and a tetracycline-regulated operator sequence, and upstream of the bovine growth hormone polyadenylation signal. The pENTR™ shuttle plasmids were used to insert either the avian H5 HA or the bovine H5 HA expression cassette into the E1 loci of the ChAdOx1 Gateway Destination plasmid (Bacterial artificial chromosome (BAC) containing ChAdOx1 genome) by Gateway recombination, as previously described [14]. Linearised viral vector genomes excised from the resulting BACs were transfected into T-REx-293 cells (Invitrogen, Birmingham, UK) using Lipofectamine 2000 (Thermo Fisher Scientific, Altrincham, UK), and the ChAdOx1 H5-Ibis and ChAdOx1 H5-Texas viral vectors were rescued and propagated. Both viral vectors were purified by CsCl gradient ultracentrifugation and titrated, as previously described [14].

### 2.4. Animals and Immunisations

Animal experiments at the Royal Veterinary College (RVC) were conducted according to the UK Animals (Scientific Procedures) Act 1986 under project licence number PP1385023, granted by the UK Home Office. Approval was also obtained from the Animal Welfare and Ethical Review Body (AWERB) at the Royal Veterinary College, approval reference number 139.

All animal experiments at the University of Oxford were conducted in compliance with the UK Animals (Scientific Procedures) Act 1986 under project licence number PP2352929, granted by the UK Home Office. Approval was also obtained from the Animal Welfare and Ethical Review Body (AWERB) at the University of Oxford.

These studies adhered to the Animal Research Reporting of In Vivo Experiments (ARRIVE) guidelines following the principles of the 3Rs (replacement, reduction and refinement).

**Figure 1:**
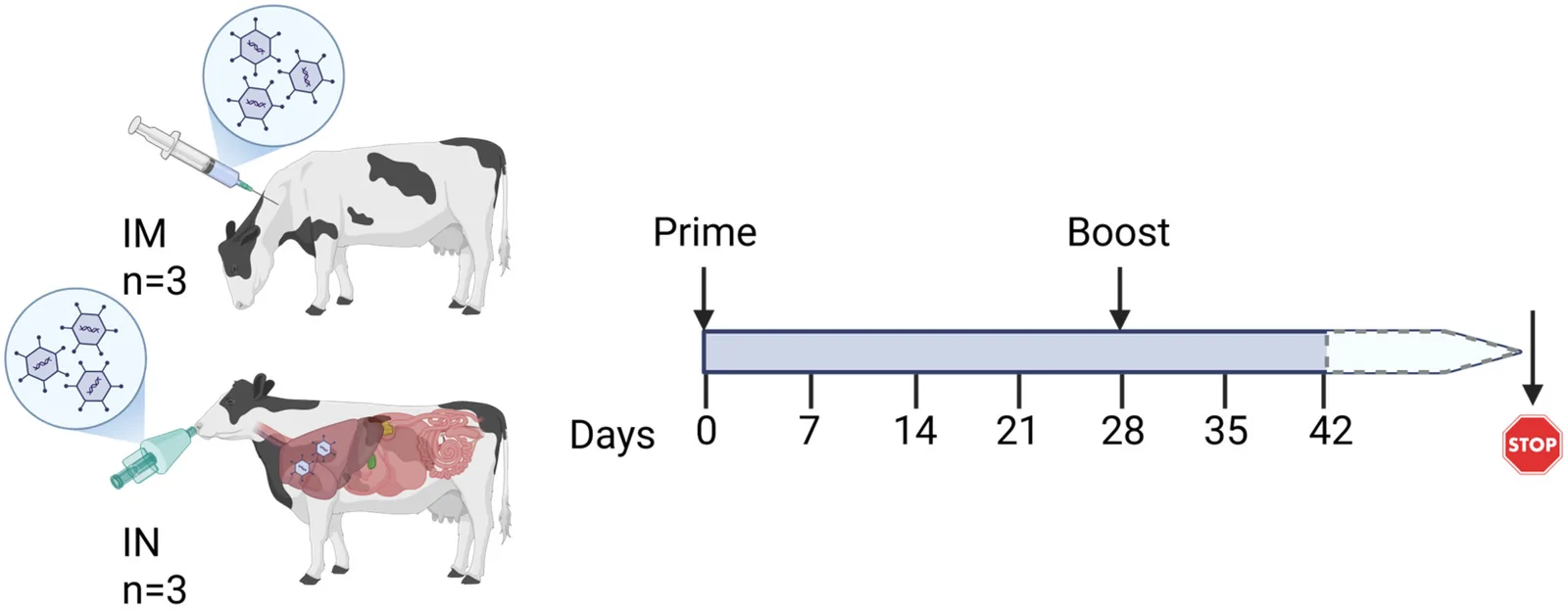
Representative illustration of the study plan carried out. Three cows were immunised either intramuscularly (IM) or intranasally (IN) with ChAdOx1 H5-Texas homologous prime-boost vaccination four weeks apart. Nine weeks after the beginning of the study, the animals were culled. Blood samples were collected weekly and milk samples three times a week. (Figure generated using Biorender.com).

At RVC: Four-year-old female Holstein Friesian cattle in mid- to late lactation were used as individual experimental units and were randomised into different groups using an online random generator. Throughout the study, cattle were housed indoors in dedicated study-yard areas within a commercial dairy herd. Apart from being milked on their own tanks and lines, with all the milk discarded as waste, cows were managed following standard practices before and during the study. Prior to starting any regulated procedures, each cow was examined by a veterinary surgeon to confirm that they were healthy and free from symptoms of disease. Cattle received a homologous prime-boost immunisation of 1 × 10^9^ IU (5.3 × 10^10^ VP) of ChAdOx1 H5 -Texas vaccine, either intramuscularly (IM; N = 3) in a total volume of 1 mL, or intranasally (IN; N = 3) in a total volume of 2 mL, 1 mL per nostril, four weeks apart. The sample size of this pilot experiment was to provide enough data for vaccine tolerability and humoral immunogenicity of the vaccination strategy, while minimising the number of animals in accordance with the principles of the 3Rs. Intramuscular immunisation was administered on the left lateral neck, and intranasal administration was achieved using the bovine intranasal RSP (Respiratory Syncytial Parainfluenza) applicator (MSD). For intranasal administration, cattle were restrained in a crush to allow safe handling of the head, and the RSP applicator was inserted into each nostril to deliver 1 mL of vaccine per nostril. Blood samples from the coccygeal or jugular vein were harvested immediately prior to vaccination and then every 7 days following the first immunisation for 6 weeks, yielding a total of 7 samples per cow. Milk samples (taken via an in-line sampling system) were collected 3 times per week from each cow at the time of milking for 6 weeks, yielding 19 samples per cow. Serum samples were heat inactivated for 30 min at 56 °C. Milk samples were centrifuged at 1500× *g* at 4 °C for 15 min to separate fat and cell debris and to clarify the fatted samples. Animals were euthanised at the end of the study (9 weeks after first immunisation) by a Schedule 1 method (Figure 1). Serum and milk samples were stored at −80 °C and delivered to Oxford University for testing and analysis. Pre-vaccination (day 0) samples from each animal were used as the baseline control to assess vaccine-induced responses over time. All the milk and serum samples were successfully collected according to the study schedule.

**Figure A1:**
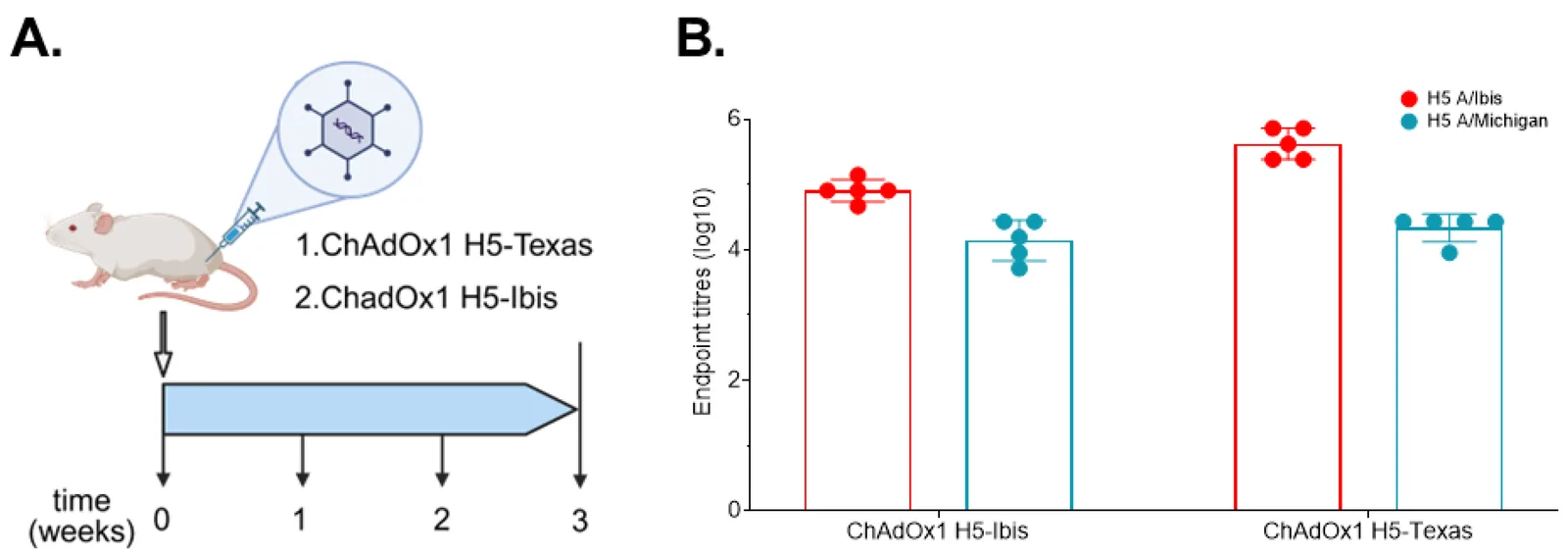
ChAdOx1 H5-Ibis and ChAdOx1-H5-Texas Influenza humoral response in mice (**A**) Experimental design; six mice were immunised intramuscularly with either ChAdOx1 H5-Texas or ChAdOx1 H5-Ibis (figure generated using Biorender.com). (**B**) Endpoint titres of IgG against either H5 from Influenza *A/Michigan/90/2024* (filled blue circle symbols) or against H5 from Influenza *A/Ibis/Egypt/RLQP-229S/2022* (filled red circles), three weeks after prime immunisation. Each symbol represents one mouse. Scatter plots with bars representing mean and standard deviations are shown.

At the University of Oxford: Mice were housed in individually ventilated cages under specific pathogen-free (SPF) conditions, maintained at a constant temperature and humidity, with a 13:11 light-dark cycle (lights on from 7 am to 8 pm). Inbred female Balb/c OlaHsd (Balb/c) mice, designated as individual experimental units, were obtained from the commercial supplier Envigo RMS UK Ltd. (Belton, UK). Upon arrival, the animals were randomly allocated into experimental groups. After one to two weeks of acclimation, 6–8-week-old mice were immunised intramuscularly (IM) in the tibialis muscle with 1 × 10^8^ infectious units (IU) of either the ChAdOx1 H5-Texas vaccine (N = 5) or ChAdOx1 H5-Ibis (N = 5), delivered in a total volume of 50 μL. Vaccine administration was carried out under general isoflurane (IsoFlo^®^) anaesthesia, ensuring full unconsciousness, verified by the absence of a pedal withdrawal reflex. Three weeks after immunisation, all mice were humanely sacrificed using an approved Schedule 1 method, i.e., exsanguination via cardiac puncture under general anaesthesia followed by cervical dislocation (Figure A1). Blood samples were harvested, and sera were used for the analysis of humoral immune responses.

### 2.5. Enzyme-Linked Immunosorbent Assay (ELISA)

For bovine samples: Humoral immune responses were evaluated using an endpoint ELISA. MaxiSorp ELISA plates were coated with 50 μL of either 2 μg/mL (IgG ELISAs) or 5 µg/mL (IgA ELISAs) H5 HA protein from either Influenza A H5N1 clade 2.3.4.4b (*A/Michigan/90/2024*, clade 2.3.4.4b, genotype B3.13 (GenoFLU)/E.2 (ggFLU)) or from Influenza *A/Ibis/Egypt/RLQP-229S/2022* (The Native Antigen, Kidlington, UK) diluted in PBS and incubated overnight at 4 °C. Following incubation, plates were washed six times with PBS containing 0.05% Tween-20 (PBS-Tween). Plates for serum samples were blocked with 100 μL per well of Blocker™ Casein (Thermo Fisher Scientific, Altrincham, UK). For milk samples, blocking was performed using casein supplemented with gelatin from cold water fish skin (Merck Group, Sigma-Aldrich, Gillingham, UK). For both sample types, blocking was performed for 1 h at room temperature. Bovine sera and milk samples were subsequently diluted in casein, and serially diluted threefold down the plate. Plates were incubated for 2 h at room temperature, washed once, then incubated for 1 h with either goat anti-bovine IgG (Thermofisher Scientific, Altrincham, UK) or sheep anti-bovine IgA (Bio-Rad, Waltford, UK) at room temperature. Following a wash step, the plates were incubated with a secondary antibody for 1 h at room temperature: donkey anti-goat IgG alkaline phosphatase (Merck Group, Sigma-Aldrich, Gillingham, UK) or donkey anti-sheep IgG alkaline phosphatase (Merck Group, Sigma-Aldrich, Gillingham, UK). Next, plates were developed by adding p-nitrophenyl phosphate (Merck Group, Sigma-Aldrich, Gillingham, UK) at 1 mg/mL in diethanolamine substrate buffer. Optical density (OD) values were measured at 405 nm using a Bio-Tek ELx800 Microplate Reader (BioTek Instruments, Winooski, VT, USA). For each sample, two technical replicates were included per plate, and mean values were used for statistical analyses. The ELISA titre was defined as the highest dilution that produced a positive signal above the mean negative control value (negative samples were composed of a pool of pre-immunisation, time 0, bovine milk or sera samples) plus three times the standard deviation. A positive control sample was used as a reaction calibrator to ensure inter-plate consistency, with a coefficient of variation below 5%. Endpoint titres were expressed as reciprocal dilutions and transformed to a logarithmic (log_10_) scale prior to statistical analysis. The limit of detection (LOD) was defined as the lowest reciprocal endpoint titre of 50, corresponding to the minimal dilution used in the assay. Samples with no detectable endpoint titre were assigned a value of 1, corresponding to a reciprocal endpoint titre dilution of 10, for statistical analysis. Statistical analyses were performed to account for repeated measurements from the same animal over time.

For mouse serum samples, an endpoint ELISA was also performed. MaxiSorp ELISA plates were coated with 50 µL of the corresponding H5 HA proteins described above, at a concentration of 2 µg/mL, and incubated overnight at 4 °C. Following a PBS-Tween wash, plates were blocked with Blocker™ Casein (Thermo-Fisher Scientific, Altrincham, UK) for at least one hour. Serial diluted (casein) serum samples were added to the plates and incubated for 2 h at room temperature. Following washing, bound antibodies were detected by incubation with alkaline phosphatase (AP)-conjugated goat anti-mouse IgG (Merck Group, Sigma-Aldrich, Gillingham, UK) for 1 h at room temperature. Plates were developed using (pNPP) substrate (Merck Group, Sigma-Aldrich, Gillingham, UK) and OD values at 405 nm were read as explained above. For each sample, two technical replicates were included per plate, and the mean value was used for statistical analysis. Historical negative control serum samples from mice enrolled in unrelated vaccine studies were used for assay development and to determine the assay cut-off to reduce the number of animals required in accordance with the 3Rs principles. A positive control sample was used as a reaction calibrator to ensure inter-plate consistency, with a coefficient of variation below 5%. The ELISA titre was defined as the highest dilution that produced a positive signal above the mean negative control value (the negative sample corresponded to a pool of historical negative control serum samples) plus three times the standard deviation. Endpoint titres are expressed as the reciprocal dilution and transformed to a logarithmic (log_10_) scale.

### 2.6. Haemagglutination Inhibition Assay (HI)

All serum and milk samples were treated with receptor-destroying enzyme (RDE, Denka-Seiken, Tokyo, Japan) to remove non-specific inhibitors. The haemagglutination inhibition (HI) assay was carried out in accordance with World Health Organization (WHO) standardised methods using 4 haemagglutination units (HAU) of virus [15]. The virus used was IDCDC-RG78A *A/American wigeon/South Carolina/22-000345-001/2021 H5N1*. For H5N1 viruses, V-bottom plates (Greiner, Dungannon, UK) were used with a 0.75% (*v*/*v*) suspension of turkey red blood cells (Biological Services, UKHSA, Colindale) in PBS. A predefined subset of samples representing the key immunological time points (pre-vaccination, day 0, post-prime, day 28 and post-boost, day 42) were selected for testing from each animal. RDE-treated milk or serum samples were used directly in the assay, with a starting dilution of 1:10. Titres are expressed as the reciprocal of the highest dilution showing complete inhibition of haemagglutination. Post-infection ferret sera raised against each test virus were included in the assay as positive controls.

### 2.7. Neutralisation Assay

All serum and milk samples were treated with receptor-destroying enzyme (RDE, Denka-Seiken, Tokyo, Japan) to remove non-specific inhibitors. The microneutralisation assay was performed using the standardised WHO protocol using virus strain (Virus IDCDC-RG78A *A/American wigeon/South Carolina/22-000345-001/2021* H5N1, as described by Lin et al. [16]. A predefined subset of samples representing the key immunological time points (pre-vaccination, day 0, post-prime, day 28, and post-boost, day 42) was selected for testing from each animal. Virus IDCDC-RG78A *A/American wigeon/South Carolina/22-000345-001/2021 H5N1* was combined with either milk or serum samples with a starting dilution of 1:10. Dilutions were added to confluent MDCK cells (Cellosaurus: CVCL_0422) in a 96-well plate. Neutralisation titres (NT_50_) are reported as the endpoint reciprocal of the highest sample dilution with a ≥50% reduction in infected cell population (ICP), relative to virus-only control. For each sample, two technical replicates were included per plate, and mean values were used for statistical analysis. The limit of detection (LOD) was defined as the minimal reciprocal endpoint titre of 10, corresponding to the minimal dilution used to perform the assay. Samples with no detectable endpoint titres were assigned an endpoint titre dilution of 5 for statistical analysis. A transformation into logarithmic (log_10_) scale has been used for representation. Statistical analyses were performed to account for repeated measurements from the same animal over time.

### 2.8. Statistical Analyses

All statistical analyses were performed using GraphPad Prism version 11.0.2 (GraphPad Software, San Diego, CA, USA). Antibody responses were analysed using a repeated-measures two-way ANOVA with Geisser–Greenhouse correction. Vaccination route was included as the between-subject factor, time as the within-subject (repeated-measures) factor, and individual cow as the repeated-measures subject. The effects of time, vaccination route, and their interaction were evaluated. Given the exploratory nature of this pilot study and the limited sample size, the results are presented descriptively.

## 3. Results

### 3.1. Evaluation of Humoral Vaccine Immunogenicity in Mice

We selected influenza *A/Ibis/Egypt/RLQP-229S/2022* as a representative of the H5N1 clade 2.3.4.4b avian influenza virus to evaluate the breadth of vaccine-induced immune responses across geographically and host-distinct viruses. Influenza *A/dairy cattle/Texas/24-008749_001/2024* also belongs to the same H5N1 clade 2.3.4.4b, but differs from Influenza *A/Ibis/Egypt/RLQP-229S/2022* by six amino acids within the HA protein. Since the Influenza *A/dairy cattle/Texas/24-008749_001/2024* vaccine was developed using influenza *A/Ibis/Egypt/RLQP-229S/2022* as a template with the introduction of targeted nucleotide substitutions, we first aimed to confirm that these mutations did not alter antigenicity and that the vaccines maintained strong cross-reactivity by determining whether ChAdOx1 H5-Texas and ChAdOx1 H5-Ibis elicited humoral immune responses in mice. Three weeks after intramuscular immunisation (Figure A1A), blood samples were harvested and the titres of IgG antibodies against H5 HA influenza *A/Ibis/Egypt/RLQP-229S/2022* and influenza *A/Michigan/90/2024* were evaluated. *A/Michigan/90/2024* was included as a more clinically relevant reference strain (CDC CL24-650397), since it shares 100% HA amino acid identity with Influenza *A/dairy cattle/Texas/24-008749_001/2024*. Comparable IgG titres were observed in both vaccine groups, indicating that the amino acid substitutions did not alter antibody recognition (Figure A1B). These findings demonstrated that ChAdOx1 H5-Texas induced a humoral immune response and elicited cross-reactive antibody responses supporting its further evaluation in dairy cattle.

### 3.2. Evaluation of Humoral Vaccine Immunogenicity in Bovine Serum

**Figure 2:**
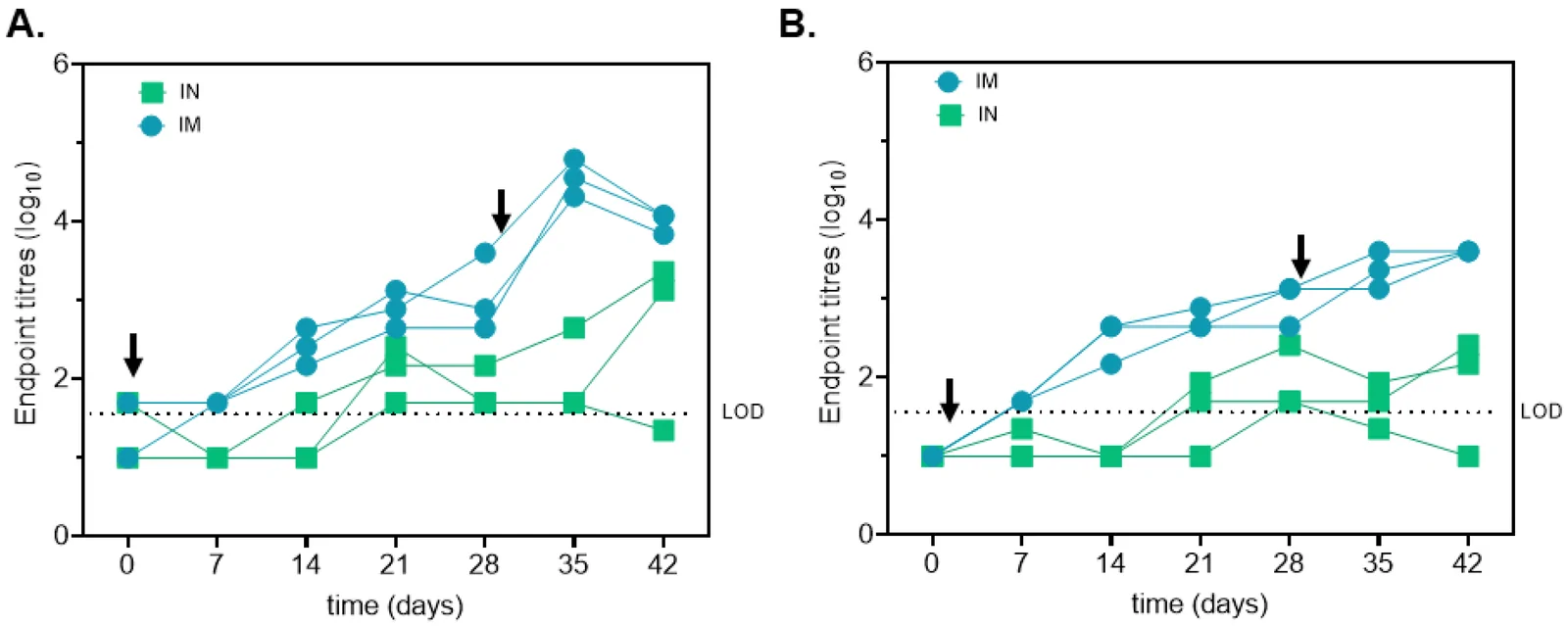
Anti-H5 HA antibody titres in sera. In-house endpoint ELISA was performed to detect IgG (**A**) and IgA (**B**) antibody titres against H5 HA from Influenza *A/Michigan/90/2024.* Blood samples were harvested every week for 6 weeks. Cows immunised with ChAdOx1 H5-Texas intramuscularly (IM) are represented with blue circles, whereas those immunised intranasally (IN) are represented with green squares. Each connected line represents one animal. Black arrows show immunisation (prime and boost) time points. Data were analysed using a repeated-measures two-way ANOVA with Geisser–Greenhouse correction. Individual animal responses are shown because of the exploratory nature of this pilot study. LOD, limit of detection.

Considering that the ChAdOx1 H5-Texas vaccine was designed to target lactating dairy cows, cattle were immunised intramuscularly, representing the conventional route of vaccination, or intranasally to induce mucosal immunity in the respiratory tract, which is the primary entry site of influenza viruses. Throughout the study, the general health and welfare of the animals was monitored by trained personnel twice daily. No animal health issues were recorded throughout the study period. Moreover, no vaccine-related adverse events were observed, including local injection site reactions, respiratory signs following intranasal administration, or changes in general health or milk production, indicating that both vaccination routes were well tolerated. Intramuscular immunisation induced higher serum titres of IgG antibodies against H5 HA of Influenza *A/Michigan/90/2024* compared to intranasal immunisation; titres exceeded the detection threshold as early as seven days post-prime immunisation (Figure 2A). In contrast, detectable levels of IgG antibodies after intranasal immunisation were not observed until three weeks following prime immunisation (Figure 2A). For intramuscular immunisation, a booster administered twenty-eight days after prime vaccination induced a peak in IgG levels (an average of 1.5-fold increase), which was detected one week later. However, titres appeared to decline at the subsequent time point (Figure 2A), likely reflecting the normal contraction phase of the humoral response, which is characterised by the loss of short-lived plasma cells and the transition to long-lived plasma cells [17]. By comparison, the intranasal booster exhibited a slower, scattered enhancement of IgG circulating titres, with two out of the three cows serologically positive at 42 days (Figure 2A).

IgA antibodies have been found to play an important role in anti-influenza immunity [18,19], and we therefore aimed to evaluate circulating IgA titres following ChAdOx1 H5-Texas immunisation. Intramuscular immunisation induced higher systemic IgA antibody titres (against H5 HA from influenza *A/Michigan/90/2024*) compared with intranasal immunisation, with detection occurring as early as seven days post primary immunisation (Figure 2B). Interestingly, IgA titres continued to increase over time without reaching a plateau, although the booster immunisation may have influenced this outcome. Intranasal immunisation induced very low circulating IgA titres that did not rise above the threshold until twenty-eight days after primary immunisation. Booster immunisation did not result in enhanced titres (Figure 2B).

**Figure A2:**
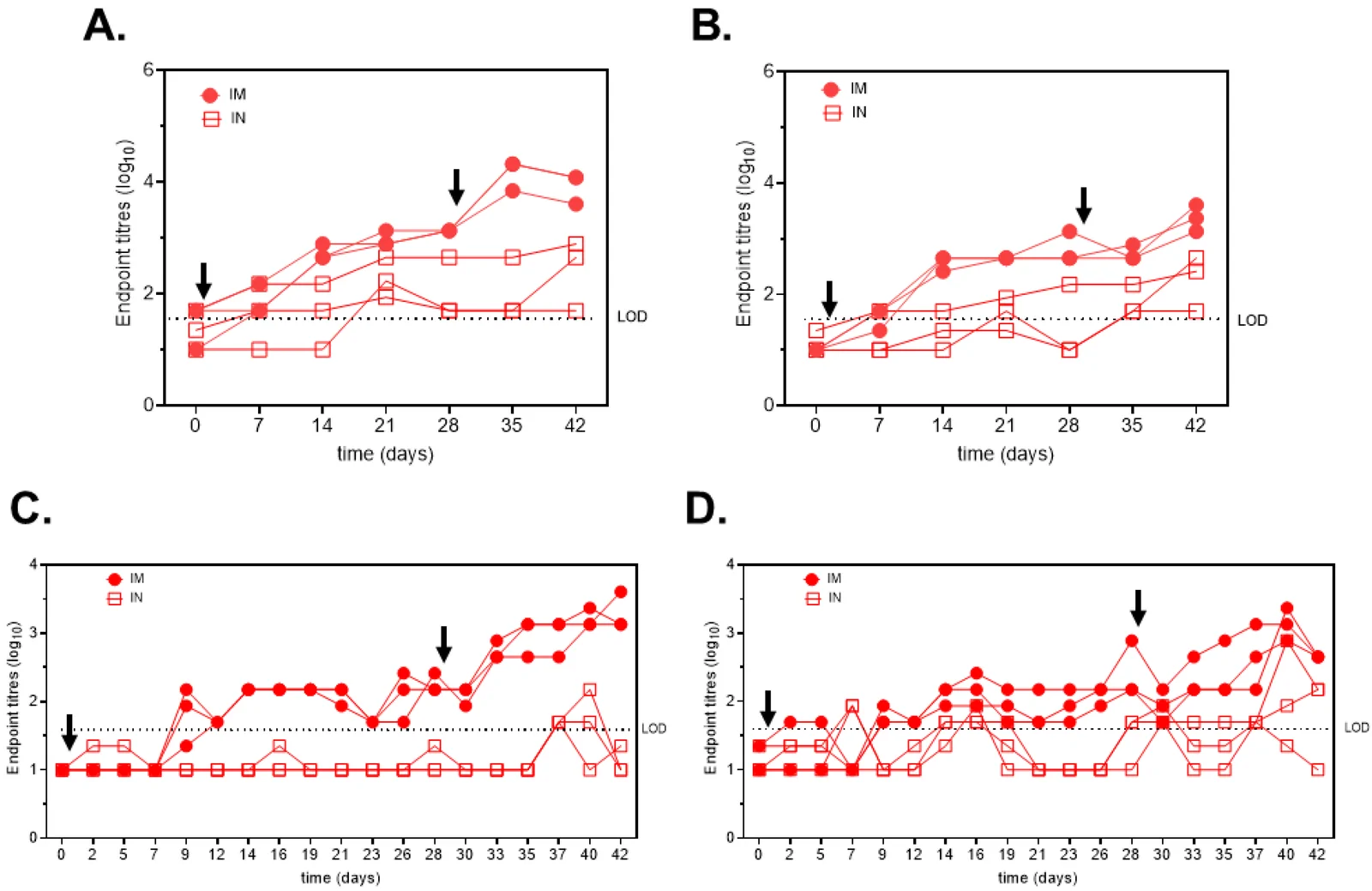
Cross-reactive anti-H5 HA antibody titres in serum and milk. In-house endpoint ELISA was performed to detect IgG (**A**,**C**) and IgA (**B**,**D**) antibody titres against H5 HA from Influenza *A/Ibis/Egypt/RLQP-229S/2022*. Blood (**A**,**B**) samples were harvested every week and milk (**C**,**D**) samples were harvested three times per week for 6 weeks. ChAdOx1-H5-Texas intramuscular (IM) immunisation is represented with red circles whereas intranasal (IN) immunisation is represented with red squares. Black arrows show immunisation (prime and boost) time points. Each connected line represents one animal. Data were analysed using a repeated-measures two-way ANOVA with Geisser–Greenhouse correction. Individual animal responses are shown because of the exploratory nature of this pilot study. LOD, limit of detection.

When antibody titres were evaluated against H5 HA from influenza *A/Ibis/Egypt/RLQP-229S/2022*, both IgG (Figure A2A) and IgA (Figure A2B) antibody titres demonstrated the same trend: *i*. intramuscular immunisation induced higher circulating antibody IgG and IgA titres than intranasal immunisation; *ii*. IgG antibodies were found in sera as early as 7 days after intramuscular immunisation, but later (21 days) after intranasal immunisation; *iii*. circulating IgA antibody titres were very low after intranasal immunisation and bordered the limit of detection; *iv*. booster parenteral immunisation induced an enhancement of IgG response and likely IgA response, whereas there was no increase following intranasal immunisation.

### 3.3. Humoral Immunity in Cattle Milk

**Figure 3:**
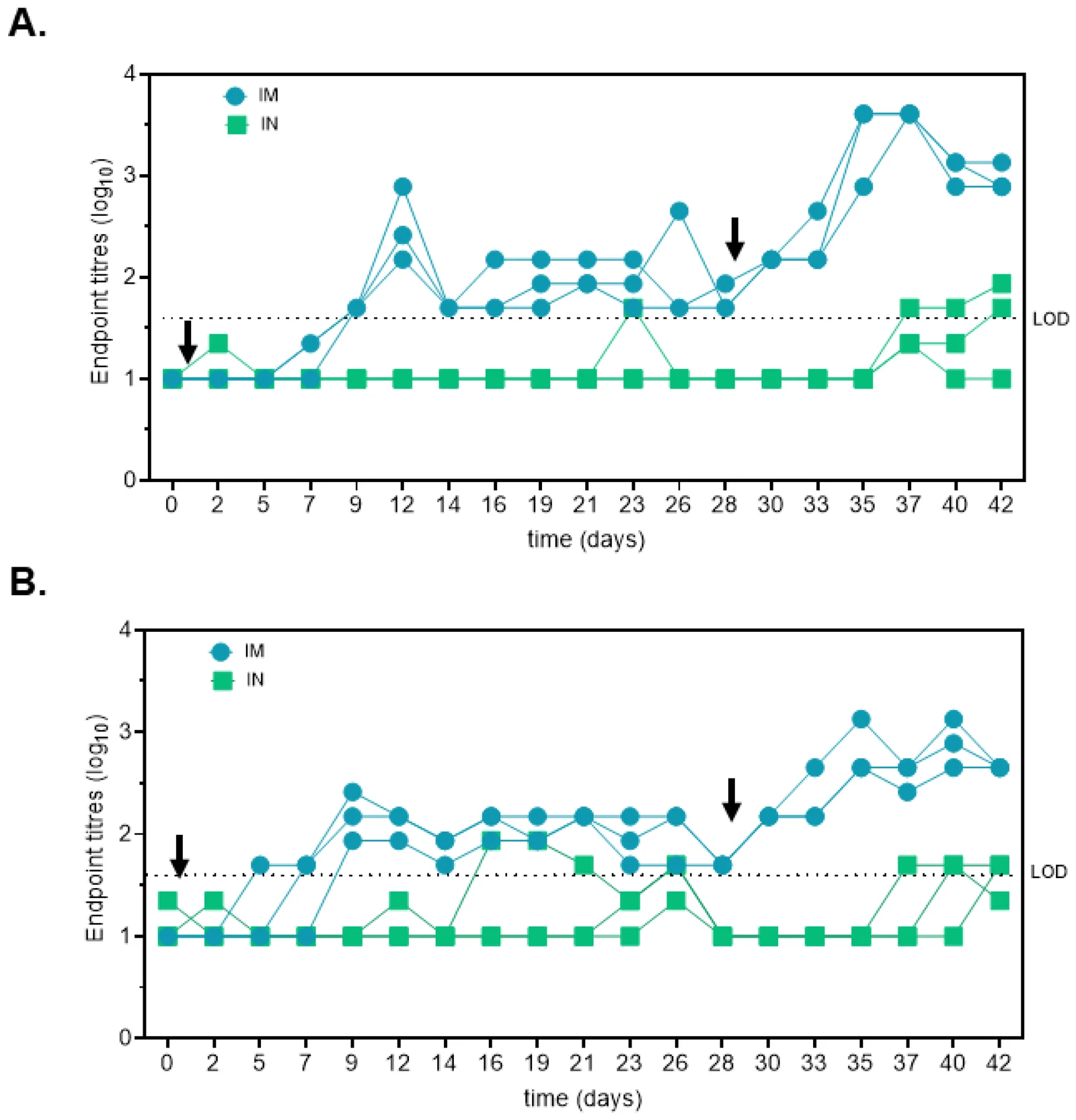
Anti-H5 HA antibody titres in milk. In-house endpoint ELISA was performed to detect IgG (**A**) and IgA (**B**) antibody titres against H5 HA from Influenza *A/Michigan/90/2024*. Milk samples were harvested 3 times a week. ChAdOx1 H5-Texas intramuscular (IM) immunisation is represented with blue circles, whereas intranasal (IN) immunisation is represented with green squares. Each connected line represents one animal. Black arrows show immunisation (prime and boost) time points. Data were analysed using a repeated-measures two-way ANOVA with Geisser–Greenhouse correction. Individual animal responses are shown because of the exploratory nature of this pilot study. LOD, limit of detection.

Clade 2.3.4.4b-specific antibodies from milk have been found to block cell entry and inhibit spread of replicating H5N1 Influenza virus in infected cows [20]. During H5N1 avian influenza virus outbreaks, milk from infected cows contained high titres of infectious virus, highlighting the concerning potential for viral spread between cows via contamination of milking equipment, as well as the risk of cattle–human transmission [21]. We aimed to evaluate whether the ChAdOx1 H5 -Texas vaccine was able to induce a humoral immune response in cow’s milk. Intramuscular immunisation induced high IgG titres against H5 HA from influenza *A/Michigan/90/2024*, which were detectable as early as 9 days after primary immunisation (Figure 3A). Booster immunisation enhanced the IgG antibody titres in milk with an average peak of approximately two-fold, 9 days after boosting. However, intranasal immunisation did not induce any detectable levels of IgG antibodies in milk, although some cows began to show borderline positivity 9 days after the booster immunisation. By day 42, corresponding to the end of the study, two out of three cows showed titres above the threshold values.

Milk IgA antibodies are involved in protecting calves from enteric pathogens, primarily via secretory IgA. However, considering their important role in neutralising influenza virus, we aimed to evaluate IgA titres in the milk. We found that parenteral immunisation induced IgA antibodies in the milk, which were detected as early as 9 days after immunisation, reflecting the pattern observed with IgG titres (Figure 3B). Moreover, booster vaccination enhanced the titres, resulting in at least a 1.5-fold increase on average by day 37 (9 days after boosting). Following the initial intranasal immunisation, no detectable levels of IgA antibodies were induced; however, boosting with a second intranasal dose had a detectable effect by day 37 in one of the cows, which became borderline positive. By day 42, two of the cows were found to be borderline positive (Figure 3B).

We also evaluated the humoral response against H5HA from influenza *A/Ibis/Egypt/RLQP-229S/2022* in milk samples and found a trend similar to that found with the H5 HA from Influenza *A/Michigan/90/2024* (Figure A2C,D): i. intramuscular immunisation induced higher IgG and IgA specific antibodies than intranasal immunisation; ii. boosting with a second parenteral dose induced an average 1.45-fold increase in IgG titres at day 42, with a maximum of an average 1.3-fold increase in specific IgA titres, which declined by day 42; iii. intranasal immunisation did not induce detectable titres of IgG and IgA in the milk. iv. Boosting intranasal immunisation with a second intranasal dose appeared to enhance IgG and IgA titres to a detectable borderline level at day 37 (9 days post-boost-immunisation), but these levels were not stable and decayed to undetectable levels.

### 3.4. Functional Role of Antibodies Detected in Sera and Milk Samples

**Figure 4:**
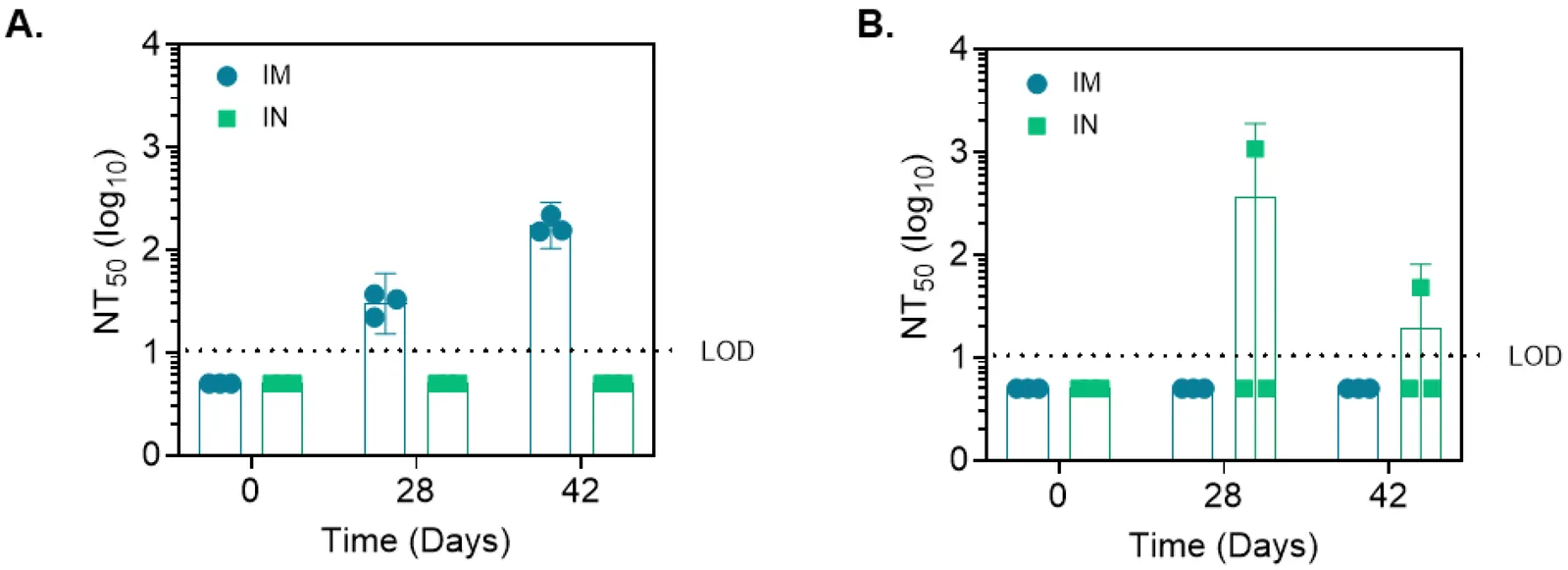
Microneutralisation assay against *A/American wigeon/South Carolina/22-000345-001/2021 H5N1* virus in cattle samples. Selected time points (day 0, day 28 (post-prime), and day 42 (post-boost)) of sera (**A**) and milk (**B**) samples were evaluated. ChAdOx1 H5-Texas intramuscular (IM) immunisation is represented with blue circles, whereas intranasal (IN) immunisation is represented with green squares. Each symbol represents one animal and bars represent the geometric mean and 95% CI for every time point. Neutralisation titre 50% (NT_50_) is represented with a logarithmic (log_10_) transformation. Data were analysed using a repeated-measures two-way ANOVA with Geisser–Greenhouse correction. Individual animal responses are shown because of the exploratory nature of this pilot study. LOD, limit of detection.

To evaluate the functional role of the antibodies detected, we assessed their ability to block influenza virus haemagglutinin from binding to sialic acid receptors using the haemagglutination inhibition assay. To focus on the most informative stages of the humoral immune response while reducing assay complexity and reagent consumption, we selected three time points for analysis: day 0, day 28 (post-prime), and day 42 (post-boost). Surprisingly, sera and milk H5-specific antibodies failed to inhibit the binding of H5N1 influenza virus (*A/American wigeon/South Carolina/22-000345-001/2021*). We next evaluated the capacity of the vaccine to induce antibodies that neutralise viral infection. Serum antibodies from vaccinated animals neutralised influenza *A/American wigeon/South Carolina/22-000345-001/2021*, a H5N1 clade 2.3.4.4b, which shares 100% HA amino acid identity with Influenza *A/dairy cattle/Texas/24-008749_001/2024,* providing a more comprehensive measure of protective antibody activity despite the absence of HI activity. Twenty-eight days after prime vaccination, intramuscularly vaccinated cows exhibited neutralising antibodies in serum, which increased by one log after a boost immunisation (Figure 4A). No neutralising titres were detected in milk samples except for a transient response in one of the three intranasally immunised cows (Figure 4B), which dropped after booster immunisation.

## 4. Discussion

The recent emergence of H5N1 clade 2.3.4.4b in dairy cattle represents a unique and concerning epidemiological event and raises alarms regarding influenza viral adaptation to a different mammalian host. In this pilot study, we have developed a ChAdOx1-vectored vaccine expressing the HA of a dairy cattle-derived H5N1 virus and demonstrated humoral immunogenicity in cattle, including the capacity to induce neutralising antibodies in serum. These preliminary findings suggest that antibody-mediated protection may not be exclusively associated with classical haemagglutination inhibition capacity. The presence of neutralising antibodies in the absence of detectable HI activity may indicate recognition of epitopes outside the haemagglutinin receptor binding site and/or alternative mechanisms of virus neutralisation that might interfere with viral attachment, membrane fusion, viral entry or other stages of replication [22]. Although additional studies are required to demonstrate the protective efficacy of the vaccine, along with the immunological mechanisms of protection, the neutralising antibody titres detected in this study were comparable to or exceeded those reported by Wiggins et al. following immunisation with a similar adenoviral-vector platform [7] and were comparable to those reported by Lee et al. using a baculoviral inactivated vaccine [23], supporting the humoral immunogenic potential of the ChAdOx1 H5-Texas vaccine in cattle.

Interestingly, respiratory infection in cows does not result in viral dissemination to mammary glands. Instead, H5N1 virus has been demonstrated to replicate efficiently within the mammary glands following oral transmission between lactating cows by cross-suckling calves (“mouth to teat transmission”) and also through fomite spread via the milking machinery, particularly the cluster, which attaches to the teats [24]. Once the virus enters the mammary glands during feeding or milking, it causes infection which may subsequently spread further within the herd [8,25]. The raw milk from infected cows may also pose a risk to dairy farm workers, in particular those milking infected cattle. The ability of the H5N1 virus to use human-type sialic acid receptors expressed in the oral tissues, teats and mammary glands of cattle [8], further highlights any zoonotic and pandemic concerns associated with ongoing adaptation to mammalian hosts.

The induction of a local immune response within the mammary glands may be of particular importance. Milk-derived antibodies and lymphocytes play an important role in the passive protection of newborn agammaglobulinemic calves and may also contribute to limiting viral replication within the mammary gland itself [26]. In our present study, we demonstrated that intramuscular vaccination is able to induce anti-H5 IgA and IgG in the milk. These antibodies may result from transference from systemic circulation in lactating cows, local production within the mammary gland, or a combination of both. Indeed, it has previously been demonstrated that plasma cell precursors from milk are not produced in the MALT (mucosa-associated lymphoid tissue) and GALT (gut-associated lymphoid tissue), but through the LN of the systemic immune system [26]. Although the present study was not designed to determine the origin of antibodies detected in the milk of vaccinated cows, further studies should focus on determining the underlying mechanism of antibody secretion into the milk by characterising immune cell populations within the mammary gland and assessing the presence of antigen-specific antibody-secreting cells.

Recognition of the HA protein of the Influenza *A/Ibis/Egypt/RLQP-229S/2022* strain by vaccine-induced antibodies in the ELISA demonstrated relevant cross-reactivity, but the functional relevance of this finding was not investigated in this study. Further evaluation with additional heterologous viruses will be performed in the future.

Although neutralising activity was not demonstrated by antibodies present in milk, the detection of vaccine-specific IgG and IgA antibodies in the milk may still be biologically relevant. Their functional significance remains unknown and requires further assessment. Previous studies have shown that protection against influenza virus is not limited to neutralising activity nor inhibition of binding to sialic acid receptors. Additional functions such as Fc-mediated effector mechanisms may also contribute to antiviral immunity [27,28]. Studies of human milk IgA have demonstrated its crucial role in killing pathogens [29] and its other antiviral properties against influenza A viruses [30], such as inhibition of viral entry and inhibition of viral release by inducing viral aggregation, as demonstrated by Yamauchi et al. [31]. In fact, IgA-mediated aggregation of influenza virions was demonstrated to reduce viral spread and enhance viral clearance [31].

A limitation of this study is the lack of evaluation of the vaccine’s protective efficacy. Although murine influenza models are commonly used for preclinical vaccine evaluation and have been used in different studies with H5N1 virus [32], they do not reproduce unique features of H5N1 infection in lactating dairy cattle, such as mammary gland involvement and virus shedding in milk. Future studies should assess the protective efficacy of the ChAdOx1 H5-Texas vaccine in challenge experiments in lactating dairy cattle, where any impact on viral shedding can be evaluated.

Following vaccination, the mammary glands from lactating cows can be seeded with antigen-specific T cells [26,33], which can then contribute to local immune protection. Several studies have demonstrated that the antigen-specific T cell repertoire plays an important role in maintaining the epithelial barrier and controlling pathogen replication at this site. Given the tropism of H5N1 viruses for the bovine mammary gland, the induction of memory T cells could represent an important mechanism to limit viral replication and shedding following infection. Adenoviral-vectored vaccines, including ChAdOx-based platforms [34], have previously demonstrated protection against influenza virus infection in a cellular-dependent manner. Although T cell responses were not evaluated in this preliminary study, the potential for the ChAdOx1 H5-Texas vector to generate antigen-specific T cell responses suggests that vaccination may provide an additional mechanism of protection beyond the humoral responses. Future studies to determine the contribution of cellular responses toward any possible protection against H5N1 infection in cattle should be conducted.

Intranasal immunisation would theoretically be one of the most appropriate routes to vaccinate against influenza virus, since it has the potential to induce a mucosal immune response at the primary site of Influenza A replication, in the respiratory tract. The intranasal immunisation was challenging to administer, but the vaccine was well tolerated, as was the intramuscular immunisation, and no vaccine-related adverse events or animal welfare concerns were raised. However, the response to intranasal immunisation in this study was disappointing, with only limited detection of H5-specific IgG and IgA antibodies in milk, and antibody titres remained at borderline levels on day 37 following booster intranasal administration of ChAdOx1 H5-Texas. Given previous studies highlighting the significance of viral shedding and spread from the udder and milk of infected cattle [8], the development of a widespread and protective local mucosal immune response would be an advantage. As a result, further optimisation of the intranasal dose and method of delivery may be worthwhile to investigate whether a better response can be achieved.

A limitation of this study is the absence of an unvaccinated or unrelated-vector control group. Therefore, in this longitudinal study, the vaccine-induced immunological responses are only compared to the individual basal levels. Moreover, another limitation of the study is the small sample size (N = 3 animals per group), which reduces statistical power and limits the precision of the effect size. We have used descriptive information and repeated measures ANOVA to assess any effects over time, and therefore the results should be interpreted with caution. Although this study shows an overall positive induction of humoral immune response over time, future studies with larger cohorts and appropriate control groups will be desirable to confirm the results.

## 5. Conclusions

We have demonstrated in this preliminary longitudinal study that the vaccine ChAdOx1 H5-Texas induces an H5-specific humoral response in serum and milk of dairy cattle. Intramuscular immunisation induced the strongest antibody responses and generated serum antibodies with neutralising capacity. Moreover, we demonstrated that the vaccine-induced antibodies exhibited binding cross-reactivity against both bovine- and avian-derived H5N1 clade 2.3.4.4b antigens, supporting the conservation of key antigenic determinants. However, the functional significance of these cross-reactive antibodies remains to be determined. Similarly, any demonstration of the protective efficacy of the vaccine against H5N1 infection, and any impact on viral shedding in cattle, are not evaluated in this study and remain to be determined in future studies. Regardless, these findings, alongside further development and evaluation, could render ChAdOx1 H5-Texas as a promising vaccine candidate for further evaluation of protective efficacy against H5N1 infection in dairy cows.

## Data Availability

The data presented in this study is available in this article, and raw data is available upon reasonable request.

## Abbreviations

The following abbreviations are used in this manuscript:

ANOVA	Analysis of variance
ARRIVE	Animal Research Reporting of In vivo Experiments
AWERB	Animal Welfare and Ethical Review Body
BAC	Bacterial artificial chromosome
ChAdOx	Chimpanzee adenovirus Oxford
CsCl	Caesium chloride
ELISA	Enzyme-linked immunosorbent assay
GALT	Gut-associated lymphoid tissue
H5HA	H5 haemagglutinin
H5N1	Haemagglutinin 5 neuraminidase 1
HAU	Haemagglutination units
HI	Haemagglutination inhibition assay
HPAI	High pathogenic avian influenza
ICP	Infected cell population
Ig	Immunoglobulin
IM	Intramuscular
IN	Intranasal
IU	Infection units
LOD	Limit of detection
MALT	Mucosa-associated lymphoid tissue
mg/mL	Milligrams per millilitre
µL	Microlitres
OD	Optical density
ORF	Open reading frame
PBS	Phosphate-buffer saline
RDE	Receptor-destroying enzyme
RVC	Royal Veterinary College
RVF	Rift Valley Fever
SPF	Specific pathogen free
USDA	US Department of Agriculture
VP	Viral particles
WHO	World Health Organisation
